# The physiological function of squalene and its application prospects in animal husbandry

**DOI:** 10.3389/fvets.2023.1284500

**Published:** 2024-01-16

**Authors:** Xin Du, Xue Ma, Yang Gao

**Affiliations:** ^1^College of Life Science, Baicheng Normal University, Baicheng, China; ^2^College of Animal Science and Technology, Jilin Agricultural University, Changchun, China

**Keywords:** squalene, oxidative stress, gut health, animal husbandry, physiological function

## Abstract

Squalene, which is a natural triterpenoid unsaturated hydrocarbon, is abundant in shark liver and plant seeds. Squalene has various physiological functions such as being anti-inflammatory and antioxidant. This paper reviews the physiological functions of squalene and its application prospects in livestock and poultry production, with a view to providing a theoretical basis for its in-depth application in animal husbandry.

## 1 Introduction

Squalene (SQ) is an unsaturated triterpene compound as well as an important component of fat-soluble vitamins, hormones, and cholesterol. In 1906, Japanese scholars extracted SQ from shark liver. Studies have shown that SQ can relieve skin damage caused by dryness and eczema; anti-oxidation (1), anti-inflammatory responses (2), and cardiovascular health are also aided by it (3, 4). A study showed that supplementing mice with SQ increased high-density lipoproteins (HDL) and paraoxonase-1 in the serum, alleviating oxidative stress damage (5). It has been shown that SQ is extremely effective in attenuating isoprenaline-induced oxidative stress in rat hearts (6). Additionally, SQ increases serum glutathione (GSH), superoxide dismutase (SOD), and catalase (CAT) levels in rats with myocardial infarction models (7). SQ research has mostly focused on production technology, material development, separation, purification, and identification in recent years (8–10). However, there are few studies on the physiological functions and mechanism of action uses of this biochemical substance in animal husbandry (1, 2). Therefore, this paper reviews its physiological functions and hypothesizes on its application prospects in animal husbandry, with a view to providing new references for green healthy farming.

## 2 The structure and properties of squalene

**Figure 1:**
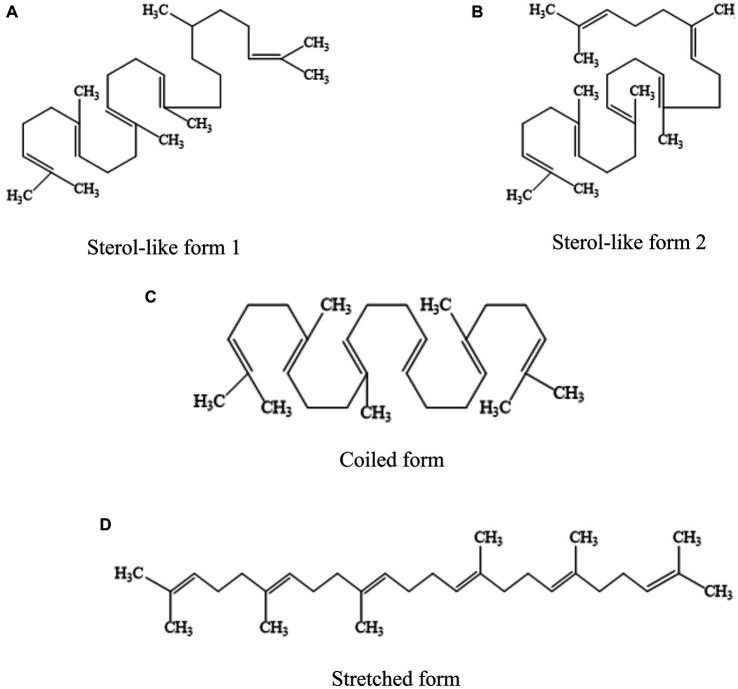
Four common structural forms of SQ. **(A)** sterol-like form 1. **(B)** sterol-like form 2. **(C)** coiled form. **(D)** stretched form.

**Figure 2:**
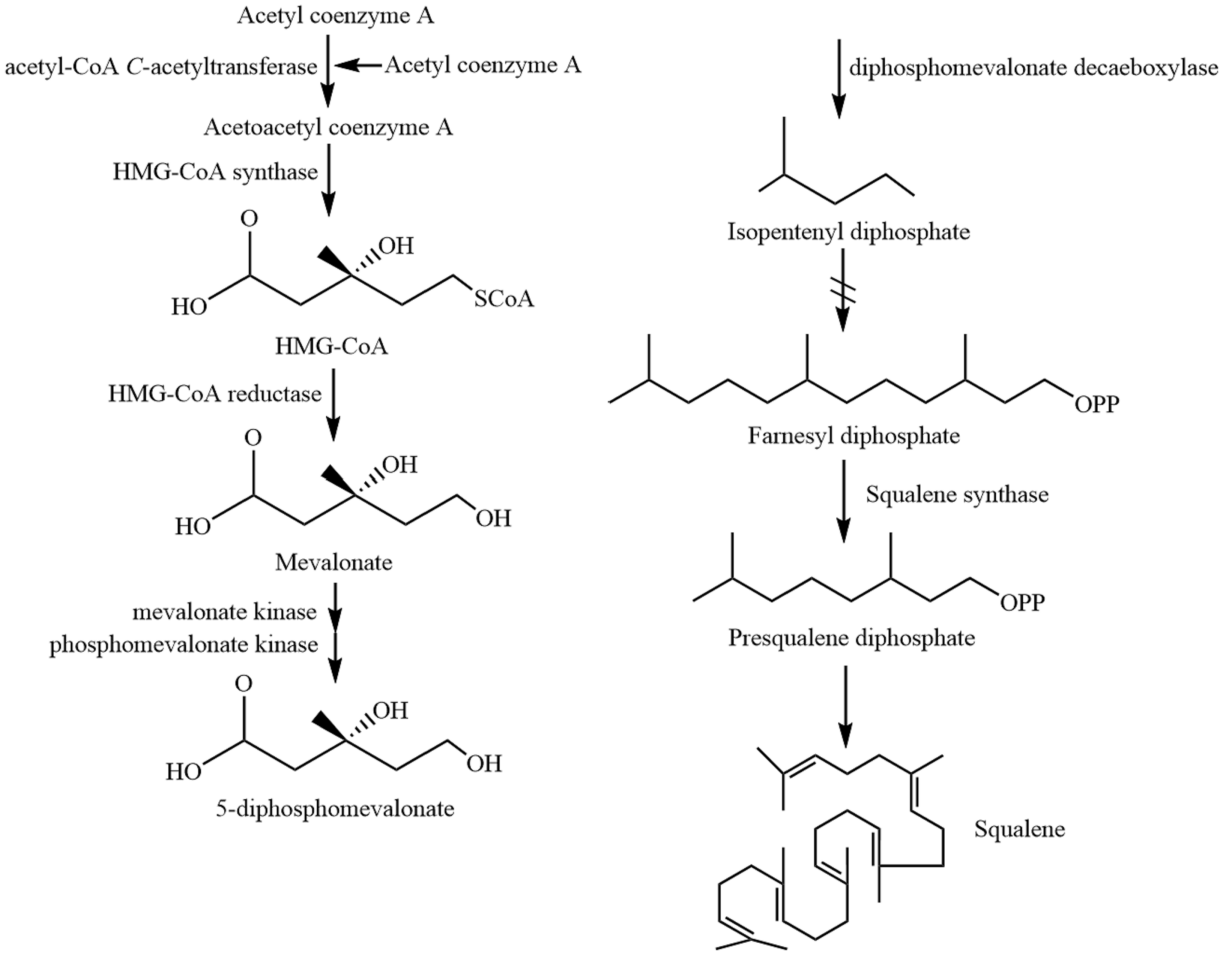
The synthesis process of SQ in animals.

Squalene (C30H50) is a non-saponifiable lipid with six double bonds. Normal SQ is a colorless, odorless liquid grease. Interestingly, all six double bonds are trans structures. As a result, SQ is less stable, oxidizes easily, and gives off a peculiar fishy smell when exposed to air for a long time. Apart from that, SQ is easy to cyclize to generate triterpenoids such as bicyclic, tetracyclic, and pentacyclic structures (4, 11). There are four main types of common SQ structures: sterol-like form 1, sterol-like form 2, coiled form, and stretched form (Figure 1). The SQ is mainly synthesized by mevalonic acid, wherein acetyl coenzyme A is first converted to 3-hydroxy-3-methylglutaryl coenzyme A (HMG CoA). Subsequently, under the influence of 3-hydroxy-3-methylglutaryl coenzyme A reductase, HMG CoA is reduced to mevalonic acid. Mevalonic acid undergoes phosphorylation and decarboxylation to generate 3-isopentenyl pyrophosphate. Ultimately, through the action of squalene synthase, two molecules of farnesyl pyrophosphate combine into an allylic moiety, leading to the formation of squalene synthase (4). The synthesis process of SQ in animals is shown in Figure 2.

In eukaryotes, SQ is an important metabolic intermediate, involved in the synthesis of animal cholesterol and plant sterols (4). It is generally believed that the unique biological characteristics of SQ are closely related to its special conformation (11). On the one hand, SQ can easily pass through the cell membrane so that it is evenly distributed inside and outside the cell (4, 11, 12). On the other hand, the abundant double bonds can combine with hydrogen ions in water to form squalane and release O2-, thus accelerating the production of new cells and improving organ function (12). Studies have shown that the antioxidant effect of SQ is related to its molecular properties and free radical activities (4). SQ can not only scavenge free radicals but also inhibit the generation of reactive oxygen species (ROS) (4). In addition, SQ, which is on the skin surface, is one of the most efficient singlet oxygen scavengers and plays an important role in resisting physical and biological stimuli (13, 14).

## 3 The physiological functions of squalene

**Figure 3:**
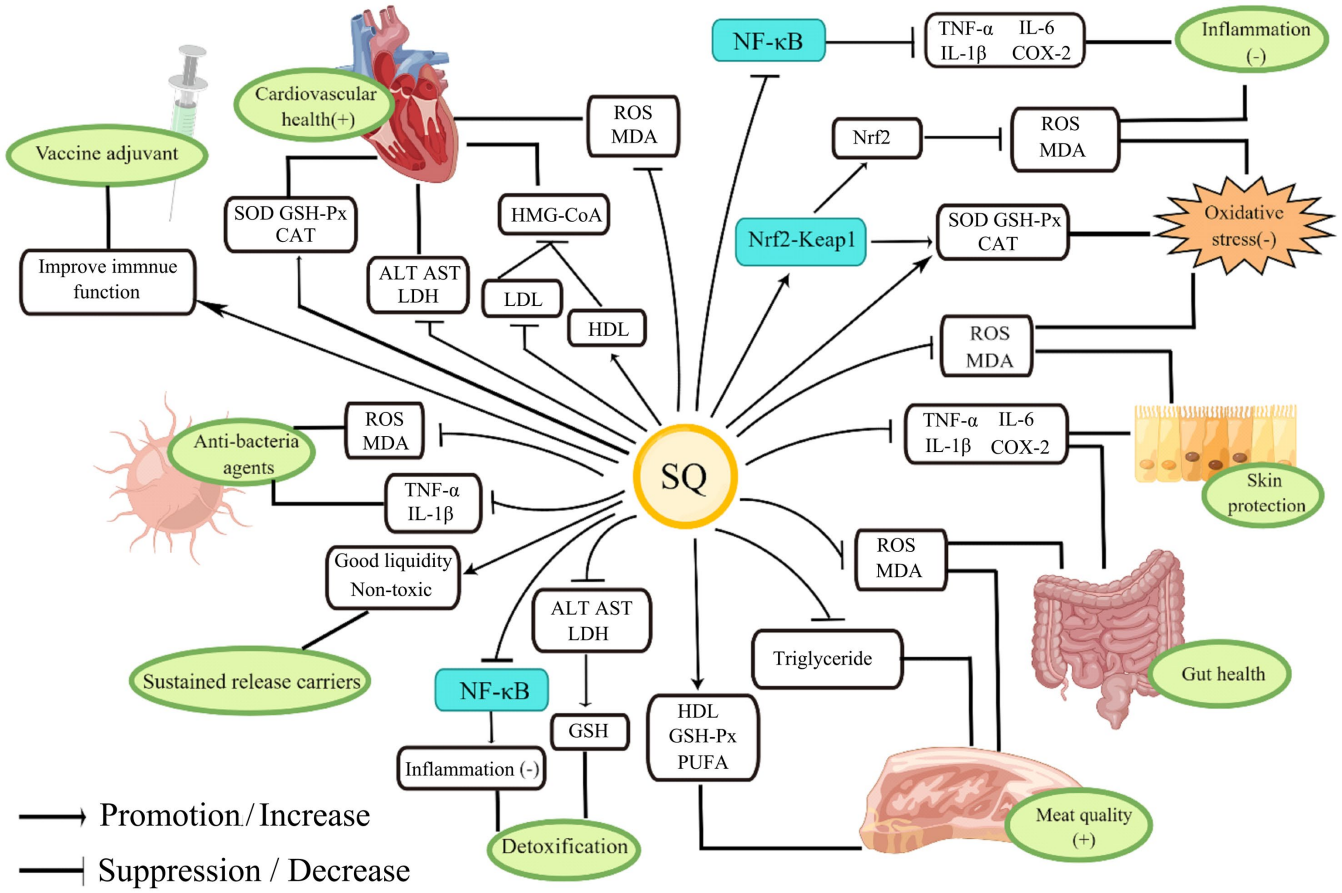
The physiological functions of SQ and its application in animal husbandry.

Squalene, as a precursor for steroid synthesis, has a variety of important biological functions such as anti-inflammatory and antioxidant properties and regulation of lipid metabolism (1–4). The physiological functions of SQ and its application in animal husbandry are shown in Figure 3.

### 3.1 Squalene and anti-inflammatory response

In the process of intensive, large-scale farming, piglet weaning, immunization, and heat stress all cause elevated ROS in the animal, which in turn produces an inflammatory response (15). An inflammatory response is a pathological process that occurs when an animal is attacked by inflammatory factors. In the case of piglets, early weaning causes elevated expression of IL-6 and IL-1β in the intestine of piglets and disrupts the intestinal structure (16). Some novel feed additives such as plant extracts and organic acids can improve the inflammatory response of animals and have important potential for application (17, 18). Research by Cardeno et al. (2) showed that SQ had great potential in regulating inflammation. SQ takes part in regulating the activation pathways of neutrophils, monocytes, and macrophages, effectively targeting anti-inflammatory factors to exert its biological functions. Studies had shown that, in a mice model of inflammation treated with liposaccharide (LPS), oral administration of 25 μm or 50 μm SQ for 18 h alleviated the inflammation effectively, mainly manifested as a down-regulation of the expression of toll-like receptor 4 (TLR-4), inducible nitric oxidesynthase gene (iNOS), cyclooxygenase (COX-2), tumor necrosis factor alpha (TNF-α), interleukin 6 (IL-6), and interferon gamma (IFN-γ) (2). By regulating the nuclear factor-kappa B (NF-κB) signaling pathway, SQ can significantly reduce the level of phosphorylated P65-NF-κB protein, regulate the expression of cellular P65 protein mRNA, and reduce the expression of heme oxygenase-1 (HO-1), which is a downstream target gene of NF-E2-related factor (Nrf2). HO-1 stimulates the production of endogenous carbon monoxide (CO), biliverdin, and iron from hemoglobin, which in turn exerts anti-inflammatory effects (19, 20). Sánchez et al. (21) demonstrated that dietary supplementation with 25 and 125 mg/kg of SQ significantly alleviated dexton sulphate sodium (DSS)-induced colitis injury in weaned mice by inhibiting the phosphor relation of the mitogen-activated protein kinases (MAPK) and NF-kB signaling pathways. This suggests that SQ reduces inflammatory cell infiltration and decreases the expression levels of TNF-α and IL-6. In addition, SQ down-regulated the expression of COX-2 and iNOS, which helped to repair the damaged intestinal mucosal epithelial barrier and reduce the inflammatory response (2, 22).

### 3.2 Squalene and oxidative stress

ROS is a one-electron reduction product of oxygen (14). Excessive ROS causes DNA, protein, and lipid peroxidation in cells, which in turn leads to cell damage, necrosis, and apoptosis (15, 23, 24). Strong oxidants, free radicals, and poisons can peroxidize cell membrane lipids, increase the permeability of cell membranes, and then cause the loss of intracellular fluid and even cell death (25). Based on their immense fluidic properties, SQ can be evenly distributed on the organ membrane structure (4, 11, 12). After membrane damage occurs, SQ can repair the injured membrane, and then exert its antioxidant effect (11, 12). The mechanism is hypothesized to occur as SQ has an abundant double-bond structure and is lipid-soluble, which rapidly fills in the damaged cell membrane structure (4). SQ can bind with hydrogen ions present in water, penetrate into cells, and enhance cellular metabolic functions (4). Studies have shown that SQ has a strong scavenging effect on singlet oxygen, and the effect is significantly higher than other lipids in the animal body (1). At the same time, SQ can significantly reduce the transmission efficiency of free radicals on the skin and protect cell DNA from damage (26). As a result, more and more studies have reported on the antioxidant properties of SQ.

Oxidative stress can cause peroxidation of LDL-C located in the inner layer of the heart, leading to increased permeability of the cardiovascular wall and damage to the structure of heart cells (27). Farvin et al. (28) showed that SQ increased the activities of antioxidant enzymes such as SOD, GSH-Px, and CAT in rat heart and alleviated isoproterenol-induced cardiac injury. Studies have shown that SQ can not only improve the antioxidant capacity of cardiovascular cells, but also maintain smooth cardiovascular epithelium, clear inflammatory reaction sites, and inhibit adhesion molecules from adsorbing low-density lipoproteins (LDL) (27). The research of Senthilkumar et al. (29) showed that, using a 150 mg cyclophosphamide-induced rat heart-injured model, daily feeding with the smallest dose of 0.4 mL SQ could alleviate oxidative damage with the highest efficiency. SQ significantly improved the functions of the enzymatic antioxidant system and non-enzymatic antioxidant system (7). Moreover, SQ effectively protected the structure of heart cells and the function of red blood cells (2, 14, 29). In a study of alcohol-induced oxidative damage, SQ also showed good antioxidant properties, mainly reflecting their protection against cell membrane lipid peroxidation (30). Aguilera et al. (31) showed that SQ could reduce alcohol-induced lipid and retinal structure damage. This is attributed to SQ’s ability to reduce the loss of polyunsaturated fatty acids, maintain normal cell membrane fluidity, and protect the optic nerve fiber layer. Thanks to research developments in recent decades focusing on the different functions of SQ, the antioxidant mechanism of SQ has been gradually discovered. Studies have shown that exogenous supplementation of SQ could significantly reduce the content of malondialdehyde (MDA) and 8-isoprostaglandin (Fα2) in animals and increase the activity of antioxidant enzymes (5). SOD plays an important role in maintaining intracellular ROS homeostasis and prevents ROS deposition in animals (23, 32, 33). MDA content reflects the degree of lipid peroxidation and the extent of biofilm damage in animals (23, 32, 33). It can be seen that SQ can maintain the redox balance in animals, scavenge excessive O2ˉ in cells, and alleviate lipid peroxidation damage.

### 3.3 Squalene and cardiovascular health

SQ can prevent abnormal elevation of glycoprotein levels in plasma or cardiac tissue, maintain GSH-Px activity, and protect cardiac tissue structure (27). In terms of improving serum lipoprotein, SQ could significantly reduce the level of LDL in the serum, improve the content of high density lipoproteins (HDL), and effectively inhibit the lipid peroxidation reaction between LDL and ROS (27). Ramírez-Torres et al. (34) pointed out that supplementing 1 g/kg of SQ daily can increase the expression of HDL-C in mouse plasma and improve the cholesterol transport capacity in plasma. Farvin et al. (35) study showed that adding 2% SQ to the diet significantly reduced levels of cholesterol, triglycerides, and free fatty acids in rat plasma and heart tissue, reducing fat deposition in the heart. It is speculated that the reason for this is that, on the one hand, SQ has lipophilicity and can synthesize stable complexes through phospholipid bilayers and bind with fatty acids, stabilizing the cellular and subcellular layers, thereby improving the antioxidant capacity of cardiovascular cells (36). On the other hand, SQ can accelerate the transport of cholesterol, triglycerides, and free fatty acids in plasma and the heart, promoting lipid metabolism (27). In addition, SQ can regulate cholesterol synthesis by inhibiting the key enzyme HMG-CoA reductase levels in the cholesterol synthesis pathway through negative feedback regulation, which is similar to the mechanism of action of statins (37). From this, it can be seen that SQ plays an important role in improving the homeostasis of the cardiovascular system’s internal and external environment, regulating cholesterol synthesis, and maintaining animal cardiovascular health.

## 4 The application prospect of squalene in animal production

### 4.1 Stress relief

When stimulated with stressors, animals will develop multiple kinds of responses, such as behavioral responses, neural responses, and endocrine responses (17). Among them, behavioral responses are the most direct way for animals to avoid stress, which can protect them from a high stress load and injury. However, in intensive production, the environment of animals is relatively closed, and the feeding mode is complex (17). Once stimulated by a stressor, such as weaning, transportation, temperature changes, vaccination, and forced molting, the behavioral response of livestock and poultry is difficult to make sense of (7). This may cause a large number of free radicals to be deposited in tissues (32, 33). When the free radicals produced by the animals cannot be eliminated, oxidative damage will occur in tissues and cells (32). In severe cases, this may lead to the death of animals. Studies have shown that supplementing SQ could effectively reduce the level of ROS in lipoproteins, and then exert antioxidant activity (2, 37). At the same time, SQ could react with the sulfhydryl group of cysteine, inhibit Nrf2 ubiquitination, and improve animal stress by regulating the Kelch-like epichlorohydrin-related protein-1 (Keap1)-Nrf2-ARE signaling pathway (2, 38, 39). The specific mechanism involves SQ promoting the dissociation of Keap1 from Nrf2, leading to the translocation of Nrf2 to the cell nucleus (38). This action enhances the expression levels of antioxidant enzyme genes such as GSH-Px, SOD, and CAT within the animal’s body, consequently reducing ROS and MDA levels (40). In addition, SQ has also been shown to have an important effect on protecting mitochondria and endoplasmic reticulum. It maintains the activity of enzymes involved in the tricarboxylic acid cycle, alters cellular energy status, and enhances the antioxidant capacity of mitochondria and the endoplasmic reticulum (41), further enhancing the resistance of animals under stress. Our research team used SQ as a feed additive for early weaned piglets. The results showed that adding 250 mg/kg SQ to the diet could effectively alleviate the stress caused by early weaning and improve production performance (42). In the poultry context, our research team discovered that dietary supplementation with 175 and 350 mg/kg of SQ significantly increased the activity of antioxidant enzymes such as SOD, GSH-Px, total antioxidant capability (T-AOC), and CAT in the serum of broiler chickens aged 1–21 days. This supplementation also notably reduced MDA levels and enhanced the antioxidant capacity of broiler chickens (43). A study has shown that SQ could significantly improve the effect on oxidative stress damage in broiler chickens caused by poisons (40). Therefore, SQ has research value when investigating it as a potential feed additive to improve the stress level of livestock and poultry.

### 4.2 Gut health

The intestine is not only the main organ for digestion, absorption, and metabolism, but also an important defense and immune barrier for the body (44). Therefore, the intestine is easily damaged by stimulation. The unique all-trans six double bond structure gives SQ excellent fluidity, which can evenly distribute and play a significant role in the mucosal layer of the intestine (4, 11, 12). In addition, SQ can fill the damaged membrane structure through cell membranes and sub-cell membranes, so to exert antioxidant and anti-inflammatory functions (4, 11, 12). Research indicates that SQ can enhance antioxidant enzyme activity in intestinal mucosa, reduce the expression levels of inflammatory factors, decrease infiltration of inflammatory cells, repair damaged intestinal epithelial barriers, and promote colonic health by inhibiting NF-κB and Nrf-Keap1 signaling pathways (21). Felices et al. (45) found that SQ could increase the expression and activity of sugar-related transporters in the intestine, promoting nutrient absorption and mitigating LPS-induced intestinal mucosal structural damage. Most piglets are weaned between 21 to 35 days old, and it takes 42 to 45 days for piglets to develop a healthy intestinal tract. In order to alleviate the intestinal damage caused by weaning, our research team added SQ to the diet of piglets weaned early between 21 to 45 days. The results showed that the diarrhea index of the piglets was significantly reduced, the permeability of the jejunum mucosa was reduced as well, and the intestinal morphology significantly improved compared with the control group (46). This could potentially be linked to the antioxidative, anti-inflammatory, and free radical scavenging properties of SQ. A study has shown that SQ can improve the antioxidant enzyme activity while removing ROS and reactive nitrogen species (RNS) (47). In the realm of intestinal microbiota, our research team discovered that dietary supplementation with 250 mg/kg of SQ significantly increased the abundance of Lactobacillus and Bifidobacterium populations and notably decreased the abundance of *Escherichia coli* populations in the cecum of early-weaned piglets (48). This helps maintain a balanced gut microbiota and promotes intestinal development. However, animal intestinal health is a complex and systematic research topic. Therefore, the mechanism of SQ to improve animal intestinal health still needs further research.

### 4.3 Skin protection

In recent animal husbandry production, while pursuing efficient development, animal welfare has gradually begun to be considered. There are often complicated conditions such as high humidity, high levels of dust, poor ventilation, and microbe and virus composition in livestock houses, and the above-mentioned conditions may have a great impact on the skin health of animals. Ozone within livestock housing can oxidize SQ present in animal skin, generating dicarbonyls and other peroxides, leading to irritation and allergic reactions, and posing substantial risks to animal skin and respiratory health (13, 49). Exudative dermatitis, skin rash, and scabies mite problems in suckling piglets and weaned piglets are mostly affected by environmental factors. Sebum plays a critical role in skin defense. SQ is an important part of sebum, with defense, moisturizing, antioxidation, and antibacterial properties (50). Studies have shown that the antioxidant capacity of SQ on the skin surface is better than that of other lipids (50). At the same time, SQ has a good singlet oxygen scavenging effect. SQ can exert anti-inflammatory and anti-oxidant effects and participate in the repair of damaged skin tissues (50). Furthermore, SQ can isolate wounds and prevent mixed infections caused by multiple harmful bacteria (51, 52), and activate macrophages and T cells, regulate the skin’s immune barrier, and can be used to treat skin conditions in piglets such as seborrheic dermatitis, rashes, and specific dermatitis (53). From this perspective, SQ, as a topical pharmaceutical agent with properties of being an antioxidant and moisturizer, not only exhibits antibacterial and anti-inflammatory functions but also possesses significant application value in safeguarding the skin and facilitating wound healing in animal husbandry.

### 4.4 Detoxification

In the process of livestock and poultry breeding, exogenous poison can be ingested into the intestinal tract, especially in free-range and grazing animals. Studies have shown that SQ is a potential detoxifier capable of removing lipophilic xenobiotics from the body. It enhances the elimination of exogenous toxins and effectively reduces their toxicity (54). Furthermore, the administration of SQ demonstrated a significant reduction in the toxicity levels of cyclophosphamide and diquat, while also restoring the blood biochemical parameters of the experimental subjects to normal levels (40). High doses of cyclophosphamide can disrupt the animal’s antioxidant enzyme system, leading to toxicity in the heart, liver, and kidneys, and ultimately resulting in multi-organ damage (40). Senthilkumar et al. (29) reported that a daily dose of 0.4 mL of SQ significantly alleviated multi-organ toxicity induced by 150 mg/kg of cyclophosphamide in rats. This treatment lowered the expression levels of injury markers such as alanine aminotransferase (ALT), aspartate transaminase (AST), and lactate dehydrogenase (LDH), while significantly increasing GSH expression compared to the control group (29). The mechanism is speculated to involve SQ’s antioxidant and membrane-stabilizing properties, which mitigate oxidative stress and tissue damage induced by cyclophosphamide (55–57). Additionally, SQ can clear toxic metabolites from the body, maintain normal antioxidant enzyme levels, and protect tissues from damage (29). Aguilera et al. (31) also found that SQ could mitigate alcohol-induced toxicity, reduce the impact of alcohol on lipid composition and structure in chicken embryo retinas, and protect the optic nerve fiber layer. Studies had shown that SQ also has a significant detoxification effect on hexachlorobiphenyl (HCB) ingested in the body (58). It not only promoted the excretion of HCB, but also reduced the absorption in the digestive tract (58). In addition, SQ alleviates the toxicity caused by heavy metals, lipopolysaccharide (LPS), pathogenic bacteria, and alkaloids (21, 33, 46). Felices et al. (45) found that SQ could alleviate the intestinal toxicity induced by LPS *in vivo* and *in vitro*. In their study, New Zealand rabbits were injected with LPS into the ear vein, and Caco-2 cells were stimulated with LPS (45). The results showed that SQ could effectively alleviate the intestinal toxicity damage by interfering with the interaction between myocin light chain kinase (MLCK) and NF-κB (45). It can be argued that SQ has great potential for application in mitigating exogenous toxicity and maintaining animal defenses.

### 4.5 Improving the quality of livestock products

Intensive and large-scale farming practices often lead to oxidative stress in livestock and poultry due to high stocking densities and poor environments, resulting in growth stagnation, pale meat, and compromised appearance (59, 60). Meat quality is one of the most important indicators in the slaughter performance of livestock and poultry, and water holding capacity is a central indicator to measure the quality of fresh meat. After slaughter, lipids and proteins in muscle are highly susceptible to oxidation, leading to the destruction of cell membranes and myofiber structures, decreased rate of protein degradation, and increased loss of moisture (61, 62). The findings of the study indicated that the inclusion of SQ in the diet of one-day-old Ross broilers had a positive linear effect on the reduction of drip loss in breast muscles. This effect was observed in order to maintain the stability of pectoral muscle pH24h and pH48h within the range of 5.8 ~ 5.9 (63). On one hand, SQ can enhance GSH-Px activity in muscles, inhibit lipid peroxidation, and improve water-holding capacity by boosting muscle antioxidant capabilities (63). On the other hand, SQ can stimulate hemoglobin production, maintaining normal muscle pH with enhancing water-holding capacity (64, 65). Additionally, a study demonstrated that adding 5% olive oil to feed reduced the content of saturated fatty acids and increased the content of polyunsaturated fatty acids in chicken breast and thigh meat, thereby improving meat quality (66). This improvement is speculated to result from the presence of abundant SQ in olive oil, which elevates serum HDL-C concentration in broiler chickens, reduces triglyceride content, and subsequently enhances meat quality. In addition to this broiler study, SQ also improves reproductive performance in boars. It was shown that the addition of SQ to the diet of 12-month-old boars significantly improved semen quality, while decreasing serum leptin levels and significantly increasing testosterone levels (67). Through calculation, it was found that SQ has the characteristics of low cost and high profit, and has great potential for application (67).

### 4.6 Other application prospects of squalene

#### 4.6.1 Vaccine adjuvant

Studies had shown that an SQ adjuvant could enhance the response speed of humoral and cellular immunity (68). Compared with conventional aluminum adjuvants, under the premise of ensuring animal safety, an SQ adjuvant produces a higher number of antibodies (≥5 times), and its immune response effect is much better. Studies have shown that using SQ to prepare inactivated oil emulsion vaccines could significantly improve the specific immune response of specific pathogen-free (SPF) chickens. At the same time, the proliferation of T lymphocytes and the transformation efficiency of spleen cells were improved (68). In production, poultry vaccines are often vaccinated in the form of nasal drops and eye drops; the pseudorabies vaccination for newborn piglets is also in the form of nasal drops. The excellent fluidity of SQ adjuvant enables the vaccine to be quickly and evenly distributed on the animal mucosal tissues, and the easy absorption of SQ also lays the foundation for an efficient immune response. Therefore, the development and utilization of SQ adjuvant in livestock and poultry production has favorable application potential and economic prospects.

#### 4.6.2 Antibacterial agents

Squalene and squalane play important roles in the immune regulation of infectious diseases (42). Nowicki and Barańska-Rybak (69) showed that shark liver oil could effectively inhibit the proliferation of bacteria and fungi during infections after skin damage. This suggests that high doses of SQ may possess antibacterial activity. SQ can regulate the activity of platelets and the synthesis of diglycerides to regulate the immune response triggered by microorganisms (70). Moreover, SQ relieves inflammation and oxidative damage by improving antigen presentation in order to inhibit the proliferation of microorganisms near the lesion (51). In addition, SQ can form a protective layer on damaged areas of the body to isolate the colonized microorganisms from contacting with oxygen. Our research team found that SQ had a significant therapeutic effect on piglet exudative dermatitis caused by a staphylococcal infection. It also significantly improved piglet diarrhea caused by weaning (42).

#### 4.6.3 Sustained release carrier

In order to increase the effectiveness of functional additives in livestock and poultry production, promote targeted delivery of additives or drugs, and prevent interference from plasma metabolism, sustained release carriers, such as montmorillonite, silk fibroin, chitosan, and SQ, have received widespread attention (71). Due to their biocompatibility, inertness, and non-toxic nature, they can form vesicles that fuse with cell membranes. Currently, SQ is widely employed as a drug delivery carrier (72). Under normal conditions, SQ is a colorless oily substance with excellent fluidity and distribution, and it can extend the half-life of a drug (72). Wang et al. showed that SQ stabilized phosphatidamide ethanolamine or copolymers through emulsion to delay the release of morphine prodrugs (72, 73). The sustained-release function of SQ lotion can be used to coat iron levoglucan injection or psoralen capsule for iron supplementation in newborn animals (51). On the one hand, SQ prolongs the half-life of the drug, improves drug potency, and reduces stress damage to newborn animals caused by multiple injections. On the other hand, the slow-release effect of SQ can reduce the total amount of drugs, reducing economic input and environmental pollution. Therefore, SQ has extremely important application prospects as a sustained-release carrier of medicine.

## 5 Conclusion

SQ has various functions such as reducing the expression level of inflammatory factors in animals, alleviating oxidative stress, detoxifying, protecting animal intestinal health, and improving meat quality. In addition, SQ can also serve as a vaccine adjuvant, antibacterial agent, and drug sustained-release carrier, which has important application potential in animal husbandry. Therefore, in the future, the application of SQ in livestock and poultry production should be increased, and its appropriate addition levels at different growth stages of livestock and poultry should be explored to promote the development of green ecological agriculture.

## Author contributions

XD: Writing – original draft, Software. XM: Conceptualization, Writing – original draft. YG: Writing – review & editing.

## Figures and Tables

**Table 1:** 

ALT	Amino transferase
AST	Aspartate transaminase
CAT	Catalase
CO	Carbon monoxide
COX-2	Cyclooxygenase
DSS	Dexton sulphate sodium
SQ	Squalene
GSH	Glutathione
GSH-Px	Glutathione peroxidase
HCB	Hexachlorobiphenyl
HDL	High-density lipoproteins
HDL-C	High-density lipoproteins-C
HMG CoA	3-hydroxy-3-methylglutaryl coenzyme A
HO-1	Heme oxygenase-1
IFN-γ	Interferon gamma
IL-6	Interleukin 6
iNOS	Inducible nitric oxidesynthase gene
Keap1	Kelch-like epichlorohydrin-related protein-1
LDH	Lactate dehydrogenase
LDL	Low-density lipoproteins
LDL-C	Low-density lipoproteins-C
LPS	Liposaccharide
MAPK	Mitogen-activated protein kinases
MDA	Malondialdehyde
MLCK	Myocin light chain kinase
NF-κB	Nuclear factor-kappa B
Nrf2	NF-E2-related factor
ROS	Reactive oxygen species
SOD	Superoxide dismutase
SPF	Specific Pathogen Free
T-AOC	Total antioxidant capability
TLR-4	Toll-like receptor 4
TNF-α	Tumor necrosis factor alpha
